# Empowering Young People Living With Juvenile Idiopathic Arthritis to Better Communicate With Families and Care Teams: Content Analysis of Semistructured Interviews

**DOI:** 10.2196/10401

**Published:** 2019-02-22

**Authors:** Stuart W Grande, Meghan R Longacre, Karin Palmblad, Meera V Montan, Rikard P Berquist, Andreas Hager, Greg Kotzbauer

**Affiliations:** 1 Division of Health Policy and Management School of Public Health University of Minnesota Minneapolis, MN United States; 2 Karolinska Institutet Department of Women and Child Health Karolinska University Hospital Stockholm Sweden; 3 Upstream Dream Bromma Sweden

**Keywords:** juvenile arthritis, interviews, health communication, patient participation

## Abstract

**Background:**

Young people living with juvenile idiopathic arthritis (JIA) face a number of communication barriers for achieving optimal health as they transition from pediatric care into adult care. Despite growing interest in mobile or wireless technologies to support health (mHealth), it is uncertain how these engagement tools might support young people, their families, and care teams to optimize preference-based treatment strategies.

**Objective:**

This study aims to examine how an mHealth patient support system (mPSS) might foster partnership between young people living with JIA, their families, and care teams.

**Methods:**

Semistructured interviews with young people (5-15 years old), their families, and JIA care teams were conducted using researcher-developed interviews guides. Transcribed data were qualitatively analyzed using conventional content analysis.

**Results:**

We conducted semistructured interviews with 15 young people, their parents, and 4 care team members. Content analysis revealed the potential of an mPSS to support productive dialogue between families and care teams. We identified four main themes: (1) young people with JIA face communication challenges, (2) normalizing illness through shared experience may improve adherence, (3) partnership opens windows into illness experiences, and (4) readiness to engage appears critical for clinic implementation.

**Conclusions:**

A human-centered mPSS design that offers JIA patients the ability to track personally relevant illness concerns and needs can enhance communication, generate consensus-based treatment decisions, and improve efficiency and personalization of care. Technology that supports continuous learning and promotes better understanding of disease management may reduce practice burden while increasing patient engagement and autonomy in fostering lasting treatment decisions and ultimately supporting personalized care and improving outcomes.

## Introduction

Young people living with juvenile idiopathic arthritis (JIA) face a number of communication barriers for achieving optimal health. JIA is one of the most common acquired chronic diseases during childhood and affects both short-term and long-term disability. JIA may develop at any age during childhood, and girls are more often affected than boys. Nomenclature as well as classification of JIA have been controversial. In an attempt to develop globally accepted terminology and criteria, the International League against Rheumatism (ILAR) introduced the term JIA [1]. Using the ILAR criteria, a Nordic population-based epidemiological study reported a JIA incidence of 15 per 100,000 [2]. JIA is not a single disease but rather a heterogeneous group of diseases, divided into seven different subgroups, within which three major onset subtypes can be identified: (1) systemic JIA*,* a systemic form with rash, fever, and commonly perimyocarditis, (2) polyarthritis with 5 or more joints involved, and (3) oligoarthritis (or pauciarticular JIA) with 4 or fewer joints involved.

Among young people, JIA can be quite painful and lasting well into adulthood. While many experience resolution of their disease, 50% will live with chronic arthritis into adulthood [3]. Although the disease is self-limiting in some children, it is not possible at the onset of disease to predict which child will recover and which will have a lifelong challenge. Early diagnosis and active therapeutic interventions are essential to minimize residual deformity and disability due to irreversible consequences of the disease such as joint destructions, asymmetric bone growth, and vision impairment. The treatment for JIA is multifaceted and requires a combination of monitoring, physical therapy, joint injections, medications, and sometimes surgery [4]. Although optimal treatment of JIA varies extensively by individual patient, making it highly preference-sensitive, research on preferences among young people living with JIA is minimal. A review of 27 studies found that children with JIA fear being seen as different from their peers and are interested in seeking health information to manage their own illness [3]. In other words, the preferences of young people reported in these studies appear focused on developing autonomy around care management [5]. For many young people who are optimistic about their future, these tools offer novel approaches that guide a process for making informed decisions about their care and social needs.

As widely recommended, the best way to determine the most appropriate treatment options, where uncertainty is high, are through assessing patient values, priorities, and experiences as part of preference elicitation [6-8]. Research suggests that children who are actively engaged in treatment consultations with parents may improve their confidence in managing JIA into adulthood [9,10]. By continuing to support children, youth, and adolescents with information, social support, and active involvement in the management of their illness, their confidence and long-term health may improve [3]. Over the last 20 years, researchers have been working on building tools to help patients with chronic conditions better communicate preferences with clinicians. Patient engagement tools like decision aids have shown that adult patients are more knowledgeable, better informed, understand their values, and may be more engaged in decision making than previously thought [11], but there is little comparable evidence for young people.

Approaches to minimize the effects of JIA on young people vary widely, and their success is often very personalized to the individual. One study evaluating a decision aid for children with JIA found that the tool had high acceptability but lower efficacy, leading authors to call for more innovative approaches to using decision aids and assessing outcomes among children [12]. A review of mobile phone and tablet apps that support personal management of illness in young people found that apps for diabetes, asthma, and chemotherapy recovery show some impact on monitoring and adherence, but the strength of evidence is weak [13]. Others examined an online decision aid for patients with rheumatoid arthritis and found that decision comfort and knowledge improved [14]. In one example, a group in Canada that designed an iPhone app for adolescents with cancer called Pain Squad+, found that applying a user-centered design strategy led to an acceptable and effective tool for the self-management of pain [15]. While evidence points to strengths of online tools for promoting self-management and guiding patient preference, there appears to be less evidence regarding online interventions or apps designed with the intention of promoting partnership, collaboration, or consensus between young people, families, and clinicians/care teams.

The benefits of using the Internet to search for information related to illness and JIA are well known, yet the documented benefits for online peer-support for young people is less robust [16]. Recent evidence of other applications for engaging young people in mobile apps for self-management demonstrate high acceptability and usability [17]. There is also evidence in other fields that peer-support has emerged naturally in online environments, where the benefits appear based on personal preferences for use, ease of access, and interaction with others who face similar challenges [18]. This suggests that online tools or apps should be meaningful to those who use them and will benefit those who take time to engage with them online [13]. The question remains how these newer technologies may best support young people to develop skills in self-management and treatment decision making that will serve them into adulthood.

In addition, developing tools to support the adolescent transition to long-term self-management in JIA are needed [19]. Research has called for shifting habits of clinician-centered problem solving to an expanded understanding of patient experience [20,21], arguably demonstrating a more general demand for co-produced care plans [22]. Conceptually, co-production reorients traditional models of care delivery towards a more patient-centered or person-centered approach. In practice, this has involved clinicians supporting a standardized assessment of patients, applying guidelines to inform care and offering non-narcotic medication management protocols [23-25]. Other patient-centered approaches include pre-visit planning by giving evidence-based information and advocacy tools to guide preference development to enhance decision making at the point of care [26]. When it comes to developing communication skills for young people as they transition from parent/clinician-supported decision-making models to more autonomous decision-making ones, the best approaches are less understood. While the challenges of communicating with young people with JIA have been well described [27], a major contributing factor to reduced quality of care outcomes for young people with JIA is poor adherence to treatment [28,29]. Adolescence is a critical time for developing long-term healthy habits as adults, and while online and mHealth skill development for self-management is suggested, there is little evidence on how best to build and evaluate these skills [27].

The purpose of this study was to examine how an mHealth Patient Support System (mPSS) could help improve health communication between young people, their parents, and their care team. This study examined how Genia might ready young people with chronic illness for their inevitable transition to adult care. Specifically, we explored how the mPSS influenced the engagement in self-management of JIA and communication between young people, their families, and their clinical care providers.

## Methods

**Figure 1:**
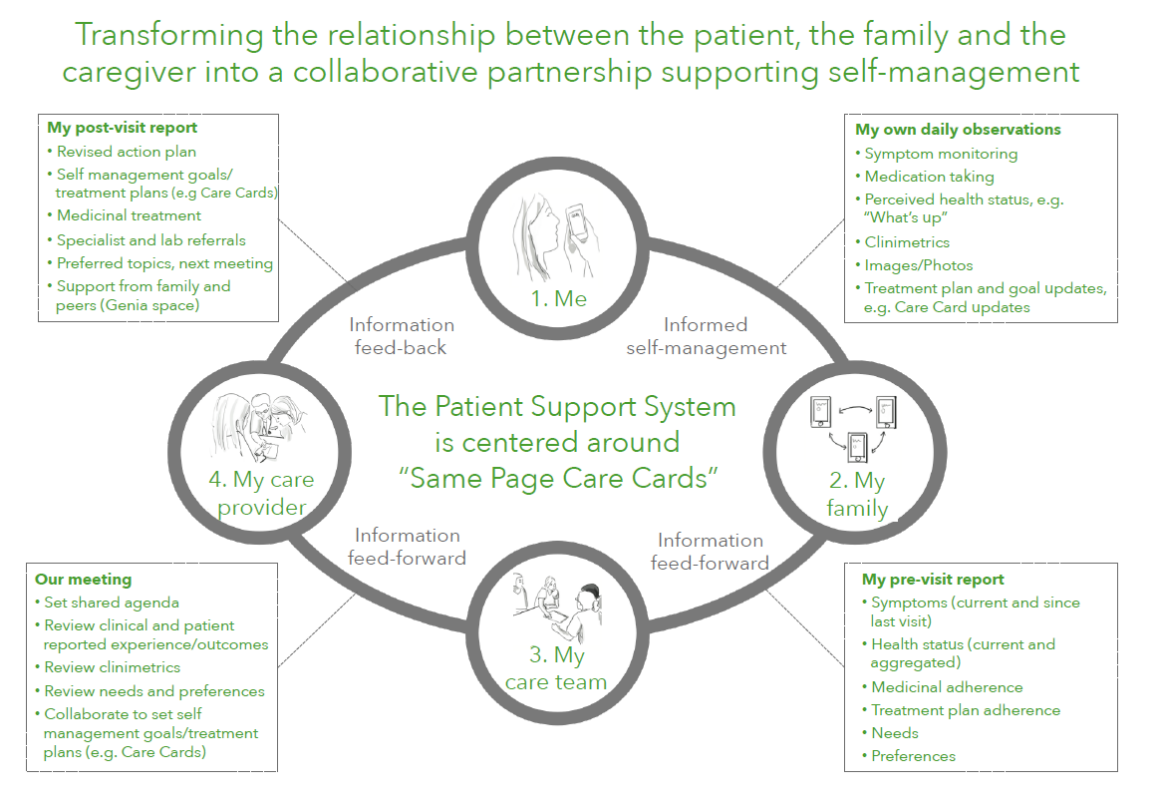
Genia flow diagram.

Genia is an iOS-based mPSS designed to establish patient, family, and care team partnerships, with an emphasis on placing the young person at the center of the decision-making process (Figure 1). As a platform and mPSS, Genia was designed with principles of user-based design [30], feed-forward systems [31], and concepts of co-production [22]. Core functionality of the mPSS were operationalized within a learning collaborative associated with the Lund Pediatric Cystic Fibrosis clinical microsystem. This meant that clinicians, researchers, family members, and cystic fibrosis patients were involved in iterative cycles of change from inception to completion of the mPSS. Key functionalities developed within the app were inspired and optimized by patients, viewed as experts in their disease and experience of care [32].

Further details of functionality are published elsewhere but summarized briefly here [32]. There is basic functionality for patient users to chat with other patient users in the app. All information entered into the app is private to users and their network. Users can, however, choose to share information with others by sending reports (ie, structured forms with specific questions for pre-visit planning) that also allow users to share self-selected daily observations and/or graphs of symptom/assessment tracking for discussion (ie, a graph of self-reported pain and fatigue in the last month). Reports can be sent through the app to clinical care teams via application program interfaces that connect to clinic IT systems. Users can also choose to send reports to others (ie, family or friends) using a basic share function within an operating system, enabling them to share via email or instant messaging.

The mPSS also captures the patient experience using a series of daily observation “trackers” that allow the patient to record daily observations and perspectives. For example, one tracker permits patients to identify what they are feeling that day, a feature notable for young people who may not be used to calling attention to or labeling their feelings. Such patterns have been identified as contributing to emotional competence in young people and are supported by parent-child and other socialization models [33]. Another tracker allows a patient to record their level of physical activity, but more unique is a tracker that includes the patient’s social interests and their assessment of life with family and with friends. This distinguishing mPSS feature permits the young person to make note of important preferences, in this case needs, wants, and fears, for subsequent review with friends, family, or care teams. This form of emotional notation and engagement has been linked to childhood development of emotional regulation and positive social engagement [34,35].

Patient-reported daily observations enable young people to document their disease activity and preferences in real-time in between clinical visits. Each of these daily data points is collected into a dashboard that can be shared electronically with a patient-determined list of observers such as family or friends they can invite to their network. They can also include these dashboards in pre-visit reports to clinical care providers. For older children and adolescents, the patient is the locus-of-control, determining who has access, the level of interaction with the app, and the amount of data being shared. For younger pediatric patients, parents can serve as proxies by entering daily observations about their child into the app.

We approached the research aim from the perspective that subjective experiences in the scope of JIA are influenced by place, persons, and institutions. For this study the place was Sweden, the persons were children aged 5-15 years living with JIA and their parents, and the place of care was the largest pediatric rheumatology clinic in Sweden, at Astrid Lindgren Children’s Hospital, within Karolinska University located in Stockholm, Sweden.

Two Swedish research assistants conducted semistructured interviews with Swedish young people, their parents, and JIA care teams about their use of and reactions to the potential benefits of the mPSS. Two researcher-designed interview guides (Multimedia Appendices 1 and 2) were developed (one for families and another for clinical team members) with clinical and project partners to include the following domains: current use of technology, health communication between clinicians and patients, personal health tracking and symptom monitoring, preparation for clinic visits, and current illness management. Data collection and analysis were supported by a multidisciplinary team, which included a medical sociologist, a health care policy expert, and a developmental psychologist. This research team worked closely with Upstream Dream, the mPSS development team in Sweden that created Genia, to gain access to clinical care partners, understand clinic organizational dynamics, and examine product development history. The study was determined to be exempt from ethics review based on Common Rule 2 by the Committee (blinded for review) for the Protection of Human Subjects at Dartmouth during a blinded review. This study was approved by the Stockholm Ethical Committee, Stockholm, Sweden. Written consent was obtained from all patients/families by the clinicians participating in the study.

### Study Participants

Potential study participants were identified by clinical partners who already use the mPSS with some of their patients and families. Clinicians then sent recruitment letters to participants with a Genia brochure inviting them to participate. The JIA clinical care team invited patients to participate in the study. Consent involved reading an informational script including the study purpose, design, and goals. Families who agreed to participate were subsequently interviewed in-person by Swedish researchers at a location and time that were convenient. Interviews were conducted with parents and children together based on preference and ethical considerations. Some of the participants had not used the mPSS prior to being interviewed, while others had already started using the mPSS. All interviews were conducted in Swedish and later translated into English. The clinical care team interviews were led by the US research team and conducted face-to-face in English at the JIA clinics in Stockholm. The clinical care team included a physiotherapist, occupational therapist, and 2 physicians of the JIA clinic. Research team members provided the interview guide to the care team members prior to the interview to give adequate time to prepare responses in English.

### Data Analysis

We conducted a conventional content analysis as described by Hsieh and Shannon [36]. Initial codes were developed independently by 2 researchers (ML and SG) using ATLAS.ti version 8.1.2. These first set of codes reflected interactions and observations by the research team from 2016-2017 as recruitment, design, and data collection were occurring. This initial code set was later updated through a second coding process based on the interview transcripts for both young people and families as well as care providers. All members of the research and clinical team who collected data spoke and wrote English fluently. To ensure accuracy of transcripts, 2 Swedish team members reviewed translations and confirmed translation with Swedish-American partners. Secondary codes were substantively related to the interview guide, in part because of how the guide was organized by domains, as well as due to the nature of the conversations that focused on specific JIA-related experiences. Following the second round of coding, the research team discussed emergent codes, which were labeled new language and concepts. Team members (SG and ML) developed memos (ie, a process of writing short descriptive narratives summarizing meaningful aspects of the data) during the coding and discussion process to draw attention to notable relationships in the codes. Team members (SG, ML, and GK) met several times over the course of 2017 to build consensus around grouping codes based on emergent and context-based themes. During the coding and subsequent consensus process, the Genia development team was asked to react to initial codes and offer alternative explanations and suggestions. Data saturation was assessed using an iterative process of constant comparison [37] throughout data collection and analysis phases. Upon a third review of data at 12 participants, the presence of language patterns and repeated concepts alerted team to potential saturation. The team agreed to three additional interviews to provide some methodological assurance that no new patient experiences were being missed or overlooked [38]. This iterative coding, memo writing, and theme building process mimics a type of data triangulation [39]. Final themes were developed by grouping codes around larger meaning units, whose content sufficiently reflected all data under consideration.

## Results

### Study Participants

The age, gender, and disease information for the study participants are shown in Table 1.

The young people interviewed for this study ranged in age from 5-15 years old. There were 7 females in the group, and 13 of the 15 had used Genia prior to the interview. The time since diagnosis ranged from 1-14 years. We also interviewed four clinicians who used Genia as part of their routine practice: 2 medical doctors, 1 physiotherapist, and 1 occupational therapist. Patient interviews were conducted with both the young person and at least one parent present. The clinician interviews were conducted individually, except two, which included both the physiotherapist and occupational therapist together.

A content analysis of transcripts generated four themes that characterize attitudes and beliefs as well as fears and expectations on how an mPSS may foster a strategy for improving communication and achieving shared decision making in JIA treatment. Experiences of young people dealing with JIA every day reflected feelings of frustration and confusion, which we termed, “Young people with JIA face communication challenges.” While the data suggest there is a developmental difference between younger and older patients’ feelings about sharing their diagnosis status with friends, there was affirming content that supported a second idea we portrayed as, “Normalizing illness through shared experience may improve adherence.” When asked about the potential of technologies or current care processes, families spoke about the value of routine check-ins between visits and symptom updates. The habit of checking-in and preparing for visits appeared to lead to deeper engagement between young people and their parents. We described this concept as, “Partnership opens a window into illness experiences.” Interview data were further informed by clinician points of view, which included support for more informed patients and families as well as for recognizing the challenge of communicating with young people. More to the point of implementation and use of a novel technology in clinical practice, we observed wide support for an mPSS and called this idea, “Readiness to engage appears critical for clinic implementation.” We present supporting illustrative quotes drawn from transcripts to characterize the categories in the sections that follow.

### Young people with Juvenile Idiopathic Arthritis face communication challenges

Young JIA patients, when they experienced pain, described feeling frustrated because people (often classmates) did not believe them, or the classmates believed they were lying to get out of an activity. This appeared to separate the young person from others, both physically and socially.

Patient 14 (14 years old): Everyone in my family is aware, so it’s okay. It’s hardest with classmates.

Interviewer: What do they say then?

Patient 14: One time there was one who said that I always blame everything on my foot.

We observed a tendency in these interviews for parents to describe interrupting the doctor’s visit to elaborate on details that either the child did not think was important or did not remember. These interruptions were viewed by young people as acceptable and often confirmatory.

Patient 7 (15 years old): I usually do it [the clinical visit] but sometimes Mom helps me to make it fair. Sometimes I say it doesn’t hurt at all, though it actually has been hurting also, then Mom jumps in [giggles].

We noticed that parents also have their own ways of dealing with their children’s pain and social struggles. Some have begun to reach out to other parents using social media platforms like Facebook, while others have talked with other parents in person. This reaching out for help reflects both the complexity and challenges of supporting children with JIA. Parents showed a desire to be helpful by wanting effective tools for their children but often lack the resources. The act of reaching out to other parents through Facebook groups underscored the importance and value of an mPSS designed to support parents to better understand their children’s illness and acquire the skills to better assist their children.

We observed young children talking about the benefits of working with their parents to help them communicate about pain and patterns in their eating and behavior. Some reflected how parents were motivated to help them develop techniques to manage their JIA independently. In one case this approach appeared to work.

Patient 10 (15 years old): Ehh...I often do not have so many of my own views. He [the clinician] usually asks me things. Sometimes I have my own questions, for example last year I had trouble with my knees, then my questions could be about that.

Mother: It’s mostly about how you’ve had it lately.

Interviewer: Do you usually go with him (asking the mother)?

Mother: Yes, I usually do. But we try to hand over it more and more to [my son] because he’s getting older. It is important now that he is moving over to adult care.

Interviewer: How do you feel about moving over to adult care?

Patient 10: Hmm...well it feels good.

**Table 1:** Description of participants.

Participant	Age in years^a^	Sex	JIA subtype^b^	Duration of disease^c^
Patient 1	11	Female	Oligo JIA	1 year
Patient 2	10	Male	PsA	4 years
Patient 3	13	Female	ERA	2 years
Patient 4	12	Male	Oligo JIA	3 years
Patient 5	11	Female	Poly JIA	1 year
Patient 6	5	Male	Oligo JIA	2 years
Patient 7	15	Female	PsA	14 years
Patient 8	12	Male	Poly JIA	4 years
Patient 9	15	Male	Undifferentiated	1 year
Patient 10	15	Male	PsA	2 years
Patient 11	11	Male	Oligo JIA	4 years
Patient 12	14	Female	JIA	5 years
Patient 13	13	Female	Oligo JIA	1 year
Patient 14	14	Female	Poly JIA	9 years
Patient 15	14	Male	JAS	4 years

^a^The average age of participants was 12.3 years (SD 2.3).

^b^Subtype for JIA: Oligo JIA=oligoarticular JIA, Poly JIA=polyarticular JIA, PsA=psoriatic JIA, ERA=enthesitis-related arthritis, JAS=juvenile ankylosing spondylitis, and undifferentiated=multisymptom JIA.

^c^The average duration of disease was 3.8 years (SD 3.5).

We further noted that most young people and adolescents in the interviews were reluctant to complain about pain or discomfort. Many felt they were a burden on their parents. Reluctance to talk may also be linked to an inability to communicate feelings effectively or how they experienced pain. As one young person showed, reluctance may be a show of strength. For example, one young person expressed his feelings and attitudes on sharing symptoms, which may have signaled strength or fear. Certainly, the interjection by the parent in the following dialogue called our attention to the challenge of communication for both parents and their children.

Patient 9 (15 years old): It feels good.

Interviewer: Do you feel that you are telling him [the clinician] exactly how you feel?

Patient 9: Yes, I think so.

Mother: ...but he always says he’s better than he is.

Patient 9: No.

Mother: Yes.

### Normalizing Illness Through Shared Experience May Improve Treatment Plan Adherence

There were some comments on connecting others with JIA through social media as a means of overcoming feelings of isolation and inability to offer help. The act of reaching out for connection may be a means of feeling normal or at least confirming that one is not alone. As we observed, younger children seemed uninterested in reaching out through social media. Yet, the fact that a 14-year old was attempting to connect virtually with others suggested that as young people age, they become more aware of their friends and social networks as sources of support.

Interviewer: Do you know how to get in touch and talk to peers?

Patient 12 (14 years old): Yes, those I’ve met I have on Snapchat and Instagram.

Interviewer: Do you have Facebook?

Patient 12: No, I don’t. But I have their numbers so I can call them.

Interviewer: The old way (ha-ha) Mother: ha-ha

Mother: (She) is in touch with some from her last rehab trip. Then also from her physical therapy, not just people with rheumatism, even people who need training.

Interviewer: How do you think it’s [nice] having friends who also have rheumatism?

Patient 12: Nice. The other day we sat up and talked and it was very nice.

We saw that young people seemed reliant on communicating with their parents, particularly when it came to navigating their relationship with their doctor or care provider. We noticed that this type of communication was not only about symptoms but about being empowered to communicate comfortably and build trust. Further, parents reflected on the power of sharing in a way their children felt confident when hearing.

Father of 11-year-old patient: Yes, absolutely. Since it is so reoccurring...and to be able to share how (she) feels, and how bad it is. If this could be possible through an app it would be great, both for us and for the doctors.

In one example, we saw parents being challenged to think about ways the mPSS may help support behaviors like monitoring pain or symptoms over time. This was seen widely as a way to improve their child’s awareness and self-management skills.

Interviewer: Do you have a diary about how you feel?

Mother: No, we do not actually. But I have it very much in my head all the time, but it’s the same all the time. But on the other hand, I think the app [Genia] can help because we do not remember the days that actually are good. It’s the misery most of the time. I think it would be great for [my daughter] to see her situation over time. So, she can see that she has better days.

The young people interviewed talked about the challenges of describing their pain and frustration with JIA, whether with physicians or friends. When asked about how Genia might contribute to talking about their JIA issues, several young people referred to the value of keeping a daily diary as a way to have better or more productive visits with their doctor.

Mother: He was so terribly afraid that it would not last (reduced pain). But then he started playing sports...living a normal life.

Interviewer: Do you remember how you felt?

Patient 15: It felt very strange at the beginning.

Mother: You came back to life.

Interviewer: Does your conversation help you manage your rheumatism?

Patient 15: Yes, it does.

Interviewer: Is there something good or less good?

Patient 15: I would say everything is good.

Interviewer: Do you keep a diary about how you feel?

Patient 15: No, only when I take syringes, then I fill it in in Genia. I think that kind of documentation is good for remembering when I talk to my doctor.

Overall feedback from young people related to the value of Genia, or potential of Genia’s functionality, suggested that diary entries and note-taking improved conversations with care teams, which helped families and young people remember benefits of certain medications and treatment decisions.

### Partnership Opens a Window Into Illness Experiences

We heard from young people and their families that using a patient support tool to keep daily observations and reminders for routine updates was very helpful for developing strategies to communicate with providers.

Interviewer: In Genia you can send a report before a health care meeting, do you think that could be something for you?

Patient 12 (14 years old): Yes, for me at least.

Interviewer: In what way?

Patient 12: I have difficulties remembering. I mix things up. I cannot tell when something was, it could have been two weeks ago or two months ago.

Mother: Although we try to be a bit prepared, it is incredibly difficult.

At the same time, clinicians also believed there was real, meaningful value in young people and their parents being better prepared for their visit.

The data is helpful, and the registry really helps me see the patient condition over time – but it only shows data at a point in time (at the time of visits). It doesn’t help me understand what is going on in the patient’s life between visits. And if they aren’t communicative during the visit, or their feelings contradict those of their parents, then it is hard for me to truly understand the patient needs; it is hard for me to understand why the patients’ perceptions of their pain and quality of life is different than my perception.

Physician 1

While having informed young people and families might improve visit efficiency by targeting interests and generating focused questions, additional behavior changes appeared to guide strategies for improved clinical conversations. The clinicians we interviewed argued that the mPSS presented a connection into the lives of patients that was meaningfully different from routine practice: “For the first time, I feel part of her [patient’s] team” (Physician 1).

### Readiness to Engage Appears Critical for Clinic Implementation

Patient monitoring following a care visit was expressed as a gap in care by clinicians. This has particular significance given that patients contend with a majority of their care challenges outside the clinic. The clinicians we interviewed shared an interest in being able to track or follow patient progress over time, which permits new and advantageous support systems: “With Genia, if we can get patients to send us status reports in between scheduled visits, then we can confirm what is working and try new ideas much more frequently” (Physician 2).

Besides the importance of tracking, clinicians reflected that engaging patients outside the clinic in meaningful and proactive ways was essential for improving communication during the clinic visit. In this way, clinicians recognized that the pre-visit data sharing feature of the mPSS app enabled more targeted and thoughtful interactions: “To improve pre-visit planning, to provide more time for dialogue with the patient during the visit, to help patients better understand what causes flare-ups and what therapy seems to work, and to provide a mechanism to track patients between visits” (Physician 1).

Other reactions highlighted the potential of the mPSS to modify routine practice. This was seen as a major improvement compared to standard care. One clinician pointed to gaps in current communication where patients are unable to speak to their symptoms, pain, or status: “When I asked how are you today, everyone says ‘fine’ but I don’t really have a clear picture. To know if there is something more I can do” (Physician 1).

Another clinician also emphasized the need for more robust efforts to guide patients prior to coming to their clinical visit. When asked if she understood the needs of her patients, one clinician suggested there was a need for improvement: “No, not really. Patients complete a long questionnaire when they get to the office, which captures responses based on industry standard instruments, but they don’t tell me [their] goals or key concerns” (Physiotherapist).

We noticed that the mPSS clarified the role of the patient, which fostered guided support and strengthened self-management skills. In fact, we saw that use of the mPSS in practice, with patients over time, appeared to change their behavior. In the case of one young person, a clinician commented that they would normally rely on the parent to share symptoms. After using the mPSS, this parent felt their child was able to do much more with the doctor: “The mother used to bring in a clipboard and do all of the talking, now the patient herself leads the conversation, leaving the mother a more bystander role” (Physician 1).

## Discussion

### Principal Findings

A co-designed mobile patient support system that meaningfully engaged users beyond the clinic visit expanded opportunities for improving treatment strategies between young people, their families, and care teams. Through the promotion of consensus-building design features, we observed improved communication in young people and their parents about symptom recognition and pain characterization. As an mHealth app, Genia, appeared to hone communication skills that parents and clinicians believe ensure a healthy transition from pediatric to adult care—a space with clear unmet need. The insights for young people, families, and clinicians to enable more substantive, targeted, and authentic communication, where young people learn to reflect on symptoms without being prompted by their parents, was a novel finding. As a mobile phone app, the routine monitoring of symptoms and pain over time normalizes a type of sharing process that appears to ease disclosure and improve efficacy in young people and parents. This was particularly relevant for young people who felt isolated by this illness and struggled to disclose their experiences. While evidence has shown how integration of novel interventions into clinical practice can be difficult, clinicians who were ready and prepared to use a novel approach appeared to mitigate barriers to Genia implementation.

### Comparison With Previous Research

With the explosive growth of and interest in mHealth initiatives, there seem to be endless opportunities for patients to engage the health system in new ways. This is one of the first studies to identify an mHealth app designed to modify communication strategies between patients, families, and care teams. Most mHealth interventions reviewed focus on self-management skill development rather than improving or optimizing patient-provider communication strategies [40]. For mHealth technology to advance behavior change strategies in young people living with JIA, more should be done to build skills that enhance consensus-building frames between patients, families, and care teams. Such an approach is consistent with the literature on the benefits of building a shared mind as a treatment strategy [41].

This study is a step towards being able to identify how an mHealth app, co-designed with patients, families, and care teams, helps identify what matters most, as well as ways of translating patient goals into actionable solutions. Focusing on principles of continuous learning and consensus building, the patient support system prioritizes processes of monitoring, reporting, and pre-visit planning to inform clinical communication. Despite the wide growth and interest in these novel technologies for improving access, there is mixed evidence on the long-term impact on patient health outcomes or behaviors [42]. This form of co-development has been well supported by health services research, which has pointed to the benefits of co-designed tools for behavior modification strategies [43-45].

A recent paper detailed the user-centered development and evaluation of an app, JIApp, which applied this same person-centered development framework and strongly supports our findings [17]. A key difference between JIApp and Genia is the co-design strategy focused on improving consensus for optimizing treatment decision making. The creators of JIApp aimed their intervention on self-management and engagement, primarily focused on “recording symptoms and encouraging self-management.” While collaboration is a key feature of patient-provider communication and self-management skills are associated with a higher quality of engagement, Genia as an mPSS was designed to modify patient-provider interaction and behaviors that likely influence consensus-building skills between young people, families, and care teams for long-term management of treatment. As mentioned previously, there is opportunity for users to chat with other users directly in the app. However, this functionality was rarely used by the participants in this study and we believe that there is more work to be done to explore how this peer-to-peer connection could be enhanced to support patients. While this is still a burgeoning area of investigation, more research is needed on understanding the co-design and implementation process of mHealth apps. Additional research is also needed to identify and measure ways that mHealth apps like Genia or JIApp modify communication responses for young people in transition from pediatric to adult care services. When compared against apps developed in isolation and often not in partnership with care providers, there is a clear benefit of co-designed apps that foster evidence-based approaches and direct input from end-users like patients and care providers [13]. Apps that support self-management and symptom management have higher potential for success, which supports the design and implementation of Genia as an mPSS [46].

What appears unique about the Genia approach in comparison to other published apps is the intentional strategy used to guide both patient and family engagement. The use of qualitative data to inform evidence on platforms that are most effective for patients has been suggested elsewhere [47]. Much of the current literature points to the strengths of mHealth to enhance self-management [42,48], tracking and monitoring [49], as well as routine behavior modification [50], and medicine adherence [42,51]. Usability testing of an online self-management health portal with adolescents with JIA and their parents underscored the importance of building these self-management platforms with user feedback, a key feature of any person-centered design [52]. Beyond the JIApp [17], there was no other evidence of mHealth apps developed to improve communication strategies or consensus-based strategies to enhance patient, family, and care team integration, particularly around the concept of shared reasoning [42]. Failures or limitations of other mHealth apps and strategies appear driven by a one-dimensional approach, which prioritizes biomarkers and tracking and fails to incorporate consensus building and communication skills. There is no question that mHealth initiatives are the way of the future; they are cheap, easy to build, and potentially highly accessible to large populations. The benefits of mHealth technology are that they are personal, adaptive, and sustainably designed. Yet, these benefits are hindered by a lack of clear standards of evaluation and measurement [53].

### Limitations

Although our study had 15 participants and involved both families and young people, the data analysis included triangulated data sources to help clarify and validate responses. Due to the number of participants and their variation in ages and experiences, findings must be read with caution. Qualitative approaches have limited generalizability outside the scope of participants’ lived experiences and other clinical settings. The unique responses of parents, care team physicians, and young people discussed here provide insight into expectations and hopes for mHealth interventions, especially interventions designed with and for end-users.

While Genia was developed in partnership with a Karolinska clinical team along with patients and parents, there were many new processes and functionalities that should be considered “new” as they were not extracted or modified from a pre-existing mobile phone app. While other research on this mPSS had been done within cystic fibrosis, the unique nature of JIA required new design and functionalities determined within a pilot phase, which has been reflected in some of the work presented here. These data and findings provide a rich and context-specific perspective of how an mPSS was adopted, integrated, and utilized by a small sample of patients and clinicians in Sweden. The nature of the research question reviewed here and its supporting methodological approach contributed to valuable context-driven findings. However, these same methods are unable to provide insight into the long-term impact of mHealth apps like Genia on changes in self-management or communication skills for young people transitioning into adult care as well among adults who have experienced transition. Moving forward, designing a study to answer long-term impacts of young people’s use of mHealth apps for self-management of JIA will be an important contribution to determine the overall efficacy of an mPSS in improving self-management and associated communication skills.

### Conclusions

A technology-enabled mPSS that meaningfully engages care providers to partner with patients, families, and their support networks permits novel care planning through the formation of a consensus-building strategy. We believe that offering patients the opportunity to engage friends, family, and their care team in developing treatment solutions may provide the emotional and clinical support they need to meet their personal health goals and sense of well-being.

## Appendix

Multimedia Appendix 1 Semistructured interview guide.

Multimedia Appendix 2 JIA care team interview guide.

## Abbreviations

ERA: enthesitis-related arthritis
ILAR: International League against Rheumatism
JAS: juvenile ankylosing spondylitis
JIA: juvenile idiopathic arthritis
mHealth: mobile health
mPSS: mHealth Patient Support System
Oligo JIA: oligoarticular JIA
Poly JIA: polyarticular JIA
PsA: psoriatic JIA
