# Supplementary material for: Empowering Young People Living With Juvenile Idiopathic Arthritis to Better Communicate With Families and Care Teams: Content Analysis of Semistructured Interviews
Source: JMIR Mhealth Uhealth. 2019 Feb 22;7(2):e10401. doi: 10.2196/10401 (PMC6406228; doi:10.2196/10401)
Supplement: Multimedia Appendix 1 [file mhealth_v7i2e10401_app1.pdf]

Semi-Structure Interview Guide

Interviewer: The first set of questions inquires about your experiences living with JIA.

1. How old were you when you were first diagnosed with JIA?

2. When you think about your experiences living with JIA, what words come to mind?

3. In your opinion, what are the biggest challenges to managing your JIA? (probe about taking medications, exercising, and communicating with family/friends)

4. Do you know how to connect and talk to others like you with JIA when you want to? (yes/no) If yes, how do you connect with others like you with JIA? How often do you talk to others like you with JIA?

5. How often do you visit a doctor for your JIA? Do you think this is too often, not often enough, or just the right number of clinic visits?

6. Describe a typical clinical visit.
    1. What do you talk about with your doctor?
    2. Does this conversation help you manage your JIA until you see your doctor again?
    3. What do you like about your visit? What don't you like?

7. Do you keep a diary about how you are doing?
    1. If no, do you think it might be helpful to keep a diary about how you are doing?
    2. Do you think it would help you if you could share certain information with your doctor shortly before your clinic visit?
        i. In your own words, how might sharing your health information with your doctor shortly before your visit improve the conversation you have about your health?

8. Thinking about your doctor visit, do you prefer to review health information together with your doctor or do you prefer to review health information on your own?

9. After your clinic visit, how well do you feel that your doctor understands your experience living with JIA? (Also probe about how well the doctor understands patient QoL and preferences for managing your JIA).

10. Describe your experience in talking about how you feel living with JIA with people in your life. (probe about family members, friends, care providers)

11. What matters most to you in being able to manage your JIA symptoms? Who are the people who matter most to you in helping you manage your JIA symptoms? How do they help?

12. In your opinion, who controls whether you feel better over time with regard to your JIA?

13. Do you have ideas about tools or methods that have helped you or could help with your JIA? Or anything else you would like to share?

C. For patients who have been using Genia for 3 or more months (February / March timeframe), and the questions we will ask at the end of the program (June / July timeframe).

Using your own words please respond to the following questions.

1. What are the biggest challenges to managing your JIA? (probe about medications, exercise.)
2. In your own words describe how you use Genia to manage your JIA.
    1. Probe about who gets to see information she provides through Genia, and what information she shares.
3. What is your primary reason for using Genia?
4. What functions of Genia do you like best, and why?
5. Are there functions that you don’t use? If not, why?
6. What is the hardest part about using Genia?
7. How has Genia changed the way you communicate with your provider(s) about JIA?

    1. Probe about whether patient feels the provider better understands her experience living with JIA, her QoL, and her preferences for managing JIA.

2. How has Genia changed the way you communicate with your family or friends about JIA?

    1. Probe about whether patient feels that family/friends better understand her experience living with JIA, her QoL, and her preferences for managing JIA.

2. Has Genia changed how frequently you would like to see your provider? If so, how?

3. Has Genia changed the way you think about your JIA? If so, how?

4. How does Genia help you think about your care options when speaking to a doctor?

5. Can you think of a better way of providing the information you capture in Genia with your doctor or care provider? Would you like to share more information you capture within or outside of Genia with your doctor or care provider either shortly prior to a visit or any time in between visits?

6. Would you recommend Genia to others with JIA? Why?
