# Supplementary material for: Empowering Young People Living With Juvenile Idiopathic Arthritis to Better Communicate With Families and Care Teams: Content Analysis of Semistructured Interviews
Source: JMIR Mhealth Uhealth. 2019 Feb 22;7(2):e10401. doi: 10.2196/10401 (PMC6406228; doi:10.2196/10401)
Supplement: Multimedia Appendix 2 [file mhealth_v7i2e10401_app2.pdf]

JIA Stockholm Interview Guide – Adopting, Using and Improving with Genia

Care Team

I. Topic: Adopting Genia – We'd like to ask you a few questions related to your adoption of Genia in your practice.

1. What were the primary reasons you wanted to use Genia?

   Probe(s):

   Describe how you might assess your patients' health between appointments?

   How fully did you understand your patients' needs before each appointment? (such as goals, prescriptions or equipment needed, lab results between visits, etc.)

   How fully did you understand your patients' needs at the end of each appointment?

   How confident were you in your ability to support your patients self-management?

2. What type of patient seemed most appropriate for using Genia? Describe this patient – age, disease severity, etc. – and why these factors influenced your selection criteria?

   Probe(s):

   Did the parent-child relationship influence your perspective when selecting the patients?

   Did the patients "adherence" to previous care plans influence your selection decision?

   After using Genia, do you still think this type of patient is most appropriate for Genia? Please explain if your perspective has changed.

3. In what ways did weekly huddles with the Genia team influence integration of Genia into the clinic care flow?

   Probe:

   In what ways have the huddles impacted your ability to use Genia effectively?

   In what ways have the weekly huddles helped or hindered continuous learning?

4. Please clarify your process for integrating Genia with patients. Have you preferred to pilot it with one patient before using with other patients? If so, can you please talk about why?

   Probe:

   When starting to use Genia, what skills (new or existing) do you think are most important? What knowledge (new or existing)?

II. Topic: Genia's Impact

Note: When answering the following questions please consider both the features of the Genia app and the support provided by the Genia team - weekly huddles or other conversations, ideas, tools.

<u>Patient Impact</u>

1. Are the patients using Genia better prepared for the clinical visit?

2. Do you think Genia improves a patient's confidence to manage and control most of their health problems?

   Probe: Do you think Genia improves a parent's confidence to help their child manage and control most of the child's health problems?

3. Do you think Genia helps patients understand their health issues?

   Probe: What Genia features lead to optimal patient self-care?

4. Does Genia help patients and parents (families) improve their shared understanding of a patient's needs and preferences?

   Probe: What Genia features lead to the improved understanding?

5. Do you think Genia has improved patients' adherence to your proposed care plan - medications or recommended therapies? One or the other?

6. Have you noticed whether Genia reduces a patient's feeling of social isolation?

<u>Care Provider Impact</u>

1. Does Genia help you learn about the things that matter most to patients about their health issues?

2. Does Genia help you include what matters most to a patient when helping them decide what to do next?

   Probe: Are there particular characteristics about a patient that typically result in Genia's positive impact?

3. Does Genia improve your satisfaction with the patient encounter?

4. Has Genia improved the efficiency of your clinical practice? The time spent with patients?

   Probe: Is Genia's data integration with the registry helpful? Is it required to achieve your goals?

<u>Care Team Impact</u>

1. Does Genia improve your relationship – coordination and communication - with your care team?

2. Has working with the Genia team helped you and your care team establish or improve your quality improvement methods?

Probe: Did you have any training in quality improvement prior to working with Genia?

III. Topic: Spreading Genia

1. How did the use of Genia spread across the clinic?

   Probe:

   a. What do you think fostered the spread from one provider to the next?
   b. What have been the main facilitators and barriers to using Genia in the clinic?
   c. Do you have to continually motivate the care team to use Genia? Patients?
   d. Based on your lessons learned with Genia thus far, what would you have done differently to introduce Genia into clinical flow? To promote the adoption of Genia by patients?
   e. What would it take to spread Genia across other JIA clinics?

IV. Topic: Wrap Up

1. How likely are you to recommend Genia to another JIA care provider? Another JIA clinic?
2. Is there anything else you would like to share? Can you think of any key improvement features (technology or process) the Genia team could implement to help you address your goals for your clinic and patients? To help patients?
